# Thermodynamic and NMR Assessment of Ligand Cooperativity and Intersubunit Communication in Symmetric Dimers: Application to Thymidylate Synthase

**DOI:** 10.3389/fmolb.2018.00047

**Published:** 2018-05-25

**Authors:** Andrew L. Lee, Paul J. Sapienza

**Affiliations:** Division of Chemical Biology and Medicinal Chemistry, UNC Eshelman School of Pharmacy, University of North Carolina at Chapel Hill, Chapel Hill, NC, United States

**Keywords:** thymidylate synthase, allostery, binding cooperativity, NMR, protein homodimer, isothermal titration calorimetry

## Abstract

Thymidylate synthase (TS) is a homodimeric enzyme with evidence for negative regulation of one protomer while the other protomer acts on substrate, so called half-the-sites reactivity. The mechanisms by which multisubunit allosteric proteins communicate between protomers is not well understood, and the simplicity of dimeric systems has advantages for observing conformational and dynamic processes that functionally connect distance-separated active sites. This review considers progress in overcoming the inherent challenges of accurate thermodynamic and atomic-resolution characterization of interprotomer communication mechanisms in symmetric protein dimers, with TS used as an example. Isothermal titration calorimetry (ITC) is used to measure ligand binding cooperativity, even in cases where the two binding enthalpies are similar, and NMR spectroscopy is used to detect site-specific changes occurring in the two protomers. The NMR approach makes use of mixed-labeled dimers, enabling protomer-specific detection of signals in the singly ligated state. The rich informational content of the NMR signals from the singly ligated state, relative to the apo and saturated states, requires new considerations that do not arise in simple cases of 1:1 protein-ligand interactions.

## Introduction

**Figure 1:**
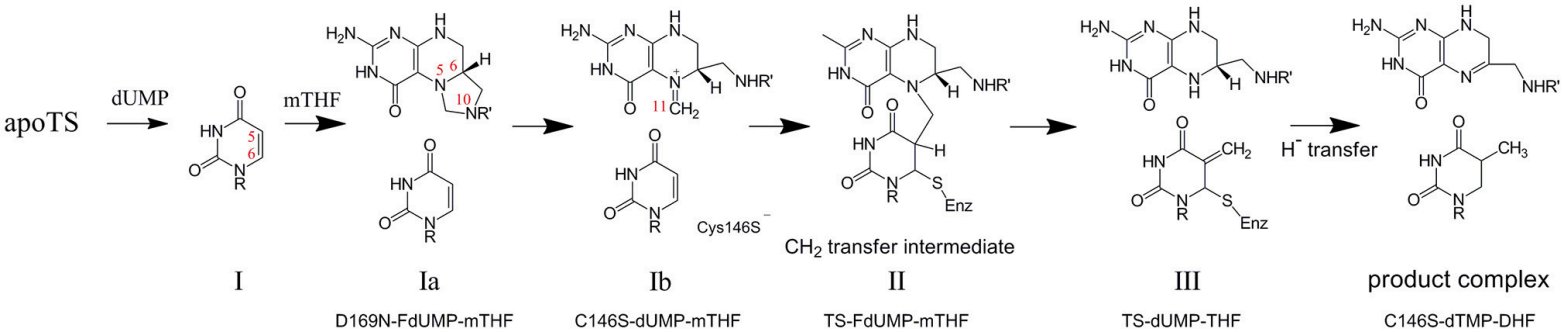
Chemical mechanism of thymidylate synthase, as determined from kinetic studies on the *E. coli* enzyme. Atom numbering is shown in red numbers. The numbering of each intermediate is in accordance with Stroud and Finer-Moore (2003), and the specific ecTS variants and ligands used to mimic those intermediates are indicated. For step Ib, “Cys146S^−^” indicates the location of residue 146 (*E. coli* TS numbering) that is mutated to serine to trap at step Ib and that in the wild-type enzyme is the active site nucleophile that bonds to C6 of dUMP.

One of the main developments in protein structural biology over the last ~15 years has been the recognition of dynamics as an important component of protein function. This is particularly evident in the case of enzymes, which have attracted much attention through a multitude of studies focused on the role of dynamics in conformational switching (Boehr et al., 2006), catalytic processes (Benkovic and Hammes-Schiffer, 2003; Henzler-Wildman et al., 2007; Nagel and Klinman, 2009; Kamerlin and Warshel, 2010; Whittier et al., 2013), and allostery (Lee, 2013; Motlagh et al., 2014; Lisi and Loria, 2016). Experimental measurement of dynamics is non-trivial, but NMR spectroscopy nevertheless represents a powerful approach to observing a range of dynamics at high resolution, and while it was initially used for study of “small” enzymes (<20 kDa) such as dihydrofolate reductase (Boehr et al., 2006), ribonuclease H (Mandel et al., 1995), and cyclophillin (Eisenmesser et al., 2005), improved NMR methods now allow work on proteins up into the hundreds of kilodaltons (Rosenzweig and Kay, 2014), thereby opening up the possibilities for NMR studies of enzyme dynamics. Our recent efforts to probe the linkage between dynamics and enzyme function have been on the homodimeric enzyme thymidylate synthase (TS) (Stroud and Finer-Moore, 2003). TS supplies the cell with deoxythymidine monophosphate (thymidylate, or dTMP) to be used in DNA synthesis, from reactants dUMP and methylene tetrahydrofolate (mTHF). In humans, TS is the target of cancer drug 5-FU and a number of other inhibitors to reduce TS activity (Phan et al., 2001; Wilson et al., 2014). The majority of enzymatic and structural studies, however, have been on bacterial forms of TS, for which a 6–9 step catalytic mechanism (Stroud and Finer-Moore, 2003; Kanaan et al., 2007) has been worked out (Figure 1).

From the many structural studies conducted by Stroud and others on TS, it is clear that the multi-step mechanism is accommodated by flexibility of the enzyme and the cofactor mTHF (Stroud and Finer-Moore, 2003). Aiding these studies is the remarkable identification of methods to trap the intermediates for structural study, either by soaking stable TS forms with substrates or through a combination of mutations and substrate analogs. This enabled “snapshots” of the reaction pathway to be captured (Stroud and Finer-Moore, 2003). Although many details are reported in the original papers (Hyatt et al., 1997; Sage et al., 1998; Stout et al., 1998; Fritz et al., 2002), overall what emerges is a picture of a TS enzyme that has the flexibility to move, in large and subtle ways, to support displacements, a cofactor ring opening, conformational reorientations of the substrates that enable binding, a methylene group transfer, and a hydride transfer toward net methylation of dUMP to dTMP. In general, TS makes a series of progressive movements through many parts of its structure to support these events, highlighted by a progressive 5 Å clamping down of a flexible C-terminus into a fixed conformation that sequesters the substrates (Kamb et al., 1992). Because these movements occur on a rapid timescale, it became clear that the conformational dynamics of TS is a critical aspect of its enzymatic function (Stroud and Finer-Moore, 2003), and it follows that investigation of these dynamics in solution by NMR is expected to increase our understanding of TS enzyme function.

**Figure 2:**
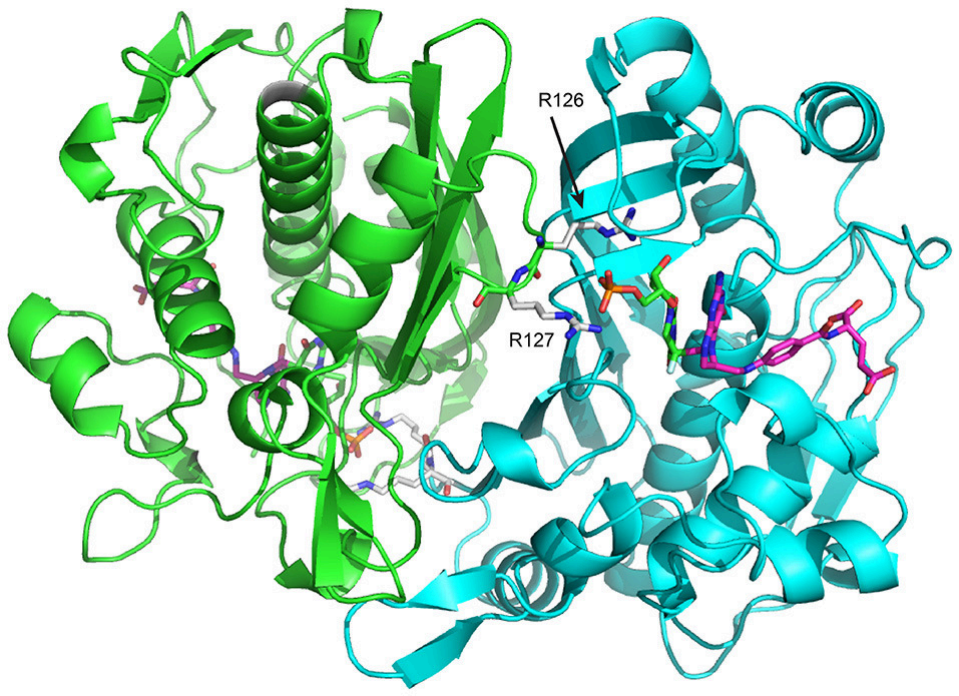
*E. coli* Thymidylate synthase (ecTS) homodimer structure with substrate and cofactor bound (“diligand,” or intermediate II in Figure 1). Each protomer is shown in green/cyan. In the cyan protomer, dUMP (carbon = green sticks) phosphate is contacted by R126 and R127 from the green protomer and is covalently attached to mTHF (carbon = magenta sticks). The pdb file used is 1TSN.

One of the fascinating properties of TS is that it has been reported to be “half-the-sites reactive,” meaning that both TS protomers cannot act on substrates at the same time (Maley et al., 1995; Johnson et al., 2002). This can be considered a form of allostery in the case of TS since the active sites are separated by 35 Å (Figure 2), although because of the various functional steps in the reaction cycle the exact point where negative cooperativity is operative is not known *a priori*. Nevertheless, it is of great interest to understand how activity in one protomer active site is communicated to the other protomer active site. The structural transitions that TS undergoes as it moves along the reaction coordinate (Stroud and Finer-Moore, 2003) are reasonable places to look for insights into interprotomer communication. However, the largest structural changes are limited to the substrate binding site and a clamping down of the C-terminus over cofactor, and the most structurally invariant regions of TS are at the extensive β-sheet rich dimer interface (Stroud and Finer-Moore, 2003), which paradoxically would seem to be the most direct route for active site-active site communication. Thus, with the exception of a unique study in which an asymmetrically bound state was crystallized for *Pneumocystis carinii* TS (discussed further below) (Anderson et al., 1999), structural analysis of TS has not led to greater insight into allosteric communication in TS. In light of the wealth of information on *Eschericia coli* TS (ecTS), in the last few years we aimed to use NMR spectroscopy as an alternative probe of intersubunit communication and to directly monitor the chemical shifts and dynamics in different functional states of the enzyme.

NMR study of TS requires consideration of spectra of dimers. Protein homodimerization has interesting consequences for the observed NMR signals, or “peaks.” The most pervasive effect is that for symmetric homodimers, the peaks for the two protomers superimpose perfectly, thus simplifying the spectrum given the overall size of the dimer. This symmetry degeneracy is often viewed as a benefit since it makes peak assignments twice as simple. By contrast, if the two protomers have structural differences, in principle the peaks from the two protomers (or subspectra) are expected to have positional differences, although an important distinction is that this will only be true in the limit of “slow exchange” on the chemical shift timescale (typically > tens of milliseconds). Thus, observation of a single set of peaks is not necessarily a guarantee of structural symmetry. In situations where information on dimer *a*symmetry is sought, distinct protomer peaks are invaluable as the protomers and their behavior can then be distinguished. It is notable that over the years the number of NMR studies on protein homodimers has been relatively low, and in the vast majority of cases the NMR information obtained was symmetric, as if the spectroscopy had only been on the monomer. One exception to this is in the cases of NMR structure determination of homodimers, where an NOE must be interpreted as arising from an intra- or interprotomer ^1^H atom, even though the two ^1^H signals have identical chemical shifts (O'Donoghue et al., 1996).

The natural tendency for homodimers to be symmetric is broken when only one binding site is filled by ligand or substrate. This sounds simple but in fact rarely occurs homogeneously in the ensemble: unless there is a great deal of negative binding cooperativity between the two (identical) binding sites in the two protomers, the second site can also become bound in a titration, in competition with binding the first of two empty sites on another dimer in the ensemble. In other words, titration with 50% ligand yields a mixture of apo (lig_0_), singly bound (lig_1_), and doubly bound (lig_2_) dimers, and hence observing the asymmetry in the singly bound state is complicated by the presence of other states and their associated signals. This is made even more complicated if there is fast exchange between species, resulting in population weighted average chemical shifts for which the weighting may not be known, making interpretation difficult. Because of these potential complications, the most convenient way to observe protomer-specific structural asymmetry effects with the atomic resolution signals of NMR is to either work on a dimer with dramatic negative cooperativity (Stevens et al., 2001; Popovych et al., 2006) or to manipulate the system to prevent binding in one of the protomers. This is the main strategy we have taken for studying intersubunit communication in TS and which will be discussed in this review.

Given the half-the-sites reactivity reported for TS, our initial strategy has been to establish TS as a system with quantifiable binding cooperativity (presumably negative) and to probe the mechanism for intersubunit communication using NMR of asymmetric, singly bound TS dimers in comparison to the apo and doubly bound forms. We have selected TS from *E. coli* (ecTS) since there are a multitude of biochemical and structural studies on this form and it yields outstanding NMR spectra for a protein of 62 kDa. The work has been primarily with two different active site binding ligands, dUMP and “diligand,” the latter of which is a covalently bound analog of dUMP substrate and mTHF cofactor that is trapped in an intermediate state of the reaction. Isothermal titration calorimetry (ITC) and NMR were used, respectively, to quantitatively probe binding cooperativity, and these approaches will be discussed in the first half of the review. The second half will focus on the NMR approach used for characterizing singly bound ecTS, the interesting peak multiplets that result from a manifold of ligand-bound states, and the findings of ecTS behavior with regard to long-range communication in these NMR studies. We finish with a discussion of consideration of using chemical crosslinks to enhance these types of studies on homo-oligomers.

## Quantifying substrate binding cooperativity in ects

**Figure 3:**
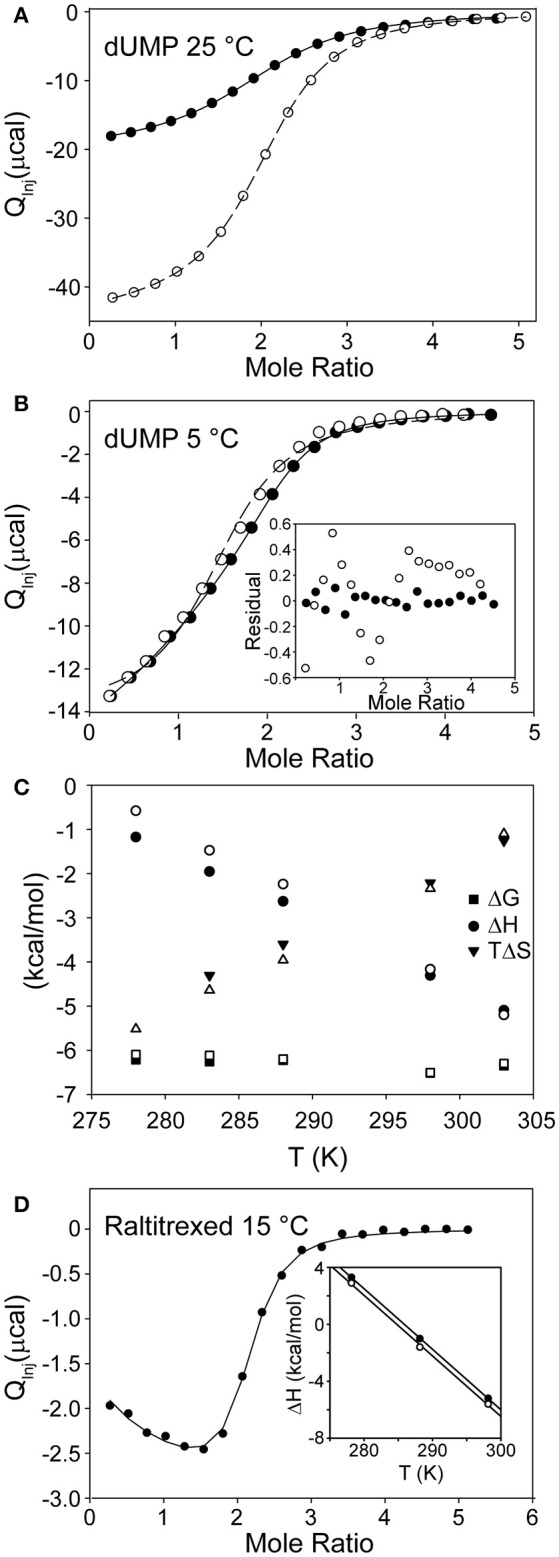
ITC of substrate and cofactor binding to TS. **(A)** Global fit of dUMP binding isotherms using two different syringe concentrations (open and filled circles) at 25°C. The modified general model (see text) was used for fitting (Sapienza et al., 2015). **(B)** Fits of dUMP binding isotherm at 5°C using two different models, the single site model with stoichiometry of two (open circles) and the modified general model (filled circles, which are shifted relative to open circles since the modified general model has cell concentration as a fitted parameter). Inset shows residuals for the two fits with modified general being clearly superior (Sapienza et al., 2015). **(C)** Thermodynamic parameters as a function of temperature for dUMP binding to free (filled symbols) and singly bound (empty symbols) TS. Note small but significant difference in slope of ΔH vs. T plots for the two binding events. **(D)** Raltritrexed binding to preformed C146S-dUMP complex at 15°C (Sapienza and Lee, 2016). Note the slope in the early part of the isotherm showing admixture of two processes with different ΔH values. Inset shows ΔH vs. T plot for first (filled circles) and second (empty circles) Raltitrexed binding events at three temperatures. Plots are parallel within error and show no signs of curvature in the narrow temperature range of measurements.

The easiest experimentally detectable explanation for half-the-sites reactivity in TS would be that dUMP substrates bind with a large degree of negative cooperativity, such that one protomer remains unbound at the cellular concentration of dUMP. A highly effective way to measure dUMP binding affinity for ecTS is to use ITC. In principle, NMR titrations could also track dUMP binding, but we found that dUMP binds with kinetics that lead to chemical exchange mostly in the fast-intermediate timescale. As a result, peaks often became faint or disappeared in the midpoints of the dUMP titration, and quantitative interpretation was difficult or impossible (Falk et al., 2016). ITC yielded high-sensitivity data that could be fitted to multiple binding models (Figure 3). The details of this were reported previously (Sapienza et al., 2015) and the salient findings are now discussed. The model that best fits the ITC data, referred to as the “modified general model” is a two-site binding model in which the two sites may have different affinities and a term is included to adjust for cell concentration (in this case, protein concentration is “modified”). Inclusion of this concentration term was crucial for accurate fitting, and without it the affinity constants can take on considerable errors (see below). For dUMP binding, using this model resulted in negligible cooperativity in ecTS at 25°C (Table 1). A convenient parameter for cooperativity is ρ = *K*_A,2_/*K*_A,1_ where the *K*_A_'s (= 1/K_D_) are the *intrinsic* association constants for the two binding sites (intrinsic *K*_A,1_ = 0.5^*^phenomenological *K*_A,1_, and intrinsic *K*_A,2_ = 2^*^phenomenological *K*_A,2_). For dUMP binding, using multiple samples, including global fits of ITC data using two cell and/or injected ligand concentrations, ρ = 0.98 ± 0.08, meaning that the intrinsic *K*_A_ values (and *K*_d_'s, which are ~17 μM) are very similar (Sapienza et al., 2015). Notably, the enthalpy changes for the two binding events are also identical at this temperature (Table 1), so in principle the single binding site model (with stoichiometry of two) could be used to fit these data. However, if the temperature is lowered to 5°C, ρ decreases to 0.80 ± 0.06 and real differences become evident in ΔH_1_ and ΔH_2_ (Table 1), necessitating the general model that allows for differences in the binding events (Figure 3B; Sapienza et al., 2015). Thermodynamic parameters based on modified general fits at different temperatures are shown in Figure 3C. ΔH vs. T plots for both binding events are linear throughout the temperature range. There is a small but significant difference in the slopes of these lines indicating different heat capacity changes for binding to the empty and singly bound protomers (${\Delta \text{C}}_{\text{P}1}^{{}^{\circ}}$ = −157 ± 1 cal/mol and ${\Delta \text{C}}_{\text{P}2}^{{}^{\circ}}$ = −183 ± 2 cal/mol). From this we conclude: (1) the two binding events are indeed different and, (2) any contributions from coupled equilibria (e.g., folding, dimerization, or ionization) are absent or minor. We cannot completely rule out coupled equilibria and an associated ${\Delta \Delta \text{C}}_{\text{P}}^{{}^{\circ}}$ because our experimental temperature range is somewhat limited, and it is unclear over which temperature range and what the magnitude of the effect would be without more knowledge (e.g., ΔH° and ${\Delta \text{C}}_{\text{P}}^{{}^{\circ}}$) about the coupled process (Eftink et al., 1983; Liu et al., 2008). However, we have shown that there is negligible proton linkage with dUMP binding at 25°C (Sapienza et al., 2015) and we can safely assert that TS remains dimeric over the entire temperature range as any change in the monomer dimer equilibrium, with the associated change in several thousand Å (Montfort et al., 1990) of solvent exposed surface area, would result in massive curvature of ΔH vs. T plots.

**Table 1:** Thermodynamic parameters for binding of dUMP to TSase^a^.

**T (°C)**	**K_1_ × 10^4^ (M^−1^)^b^**	**K_2_ × 10^4^ (M^−1^)**	**Δ${\text{H}}_{1}^{{}^{\circ}}$ (kcal/mol)**	**Δ${\text{H}}_{2}^{{}^{\circ}}$ (kcal/mol)**	**ρ**
5	7.7 ± 0.8	6.2 ± 1.0	−1.2 ± 0.01	−0.58 ± 0.01	0.80 ± 0.06
25^c^	6.0 ± 0.1	5.9 ± 0.1	−4.5 ± 0.01	−4.4 ± 0.02	0.98 ± 0.08

a *Data from Sapienza et al. (2015), Conditions are 25 mM NaPO4, 1 mM EDTA, 2 mM TCEP*.

b *Intrinsic binding constants from fits to the modified general model*.

c *Mean and standard deviations from multiple samples; including global fits of multiple c-value datasets (e.g., Figure 3A)*.

In a later study, this behavior was even more enhanced for binding of Raltitrexed, an analog of cofactor mTHF, at 15°C (Figure 3D), where ΔH_2_ is 60% larger than ΔH_1_. In this case, the different enthalpies of binding can be seen by eye in the ITC isotherm, indicating that the binding events are indeed inequivalent. In that study, the mutant C146S was used to eliminate covalent bond formation (Sapienza and Lee, 2016). At 25°C the Raltitrexed binding isotherm shows no indication of cooperativity. Further, the ΔH vs. T plots are linear and essentially parallel for the first and second binding events over the more limited temperature range (5–25°C) of these measurements. The linearity of these plots suggest similar processes associated with both binding events and that there are no linked equilibria, bearing in mind the caveats discussed above for dUMP binding. Thus, for both dUMP and Raltitrexed binding, while in a general or biologically relevant sense the degree of binding cooperativity is essentially zero, quantitatively there is “a touch” of allostery at certain temperatures and hence formally there appears at least to be some capacity for interprotomer binding cooperativity in ecTS when one considers closely the thermodynamics of binding. As a final note, we have found from multiple ITC experiments that the appearance of the isotherms can be deceptive at times. Simulations show that there can indeed be substantial degrees of cooperativity with no visible trace in the isotherms. It is therefore advisable that global fits be carried out on ITC data using different c values (Freiburger et al., 2015; Sapienza et al., 2015), different temperatures (Freiburger et al., 2012), or both.

**Figure 4:**
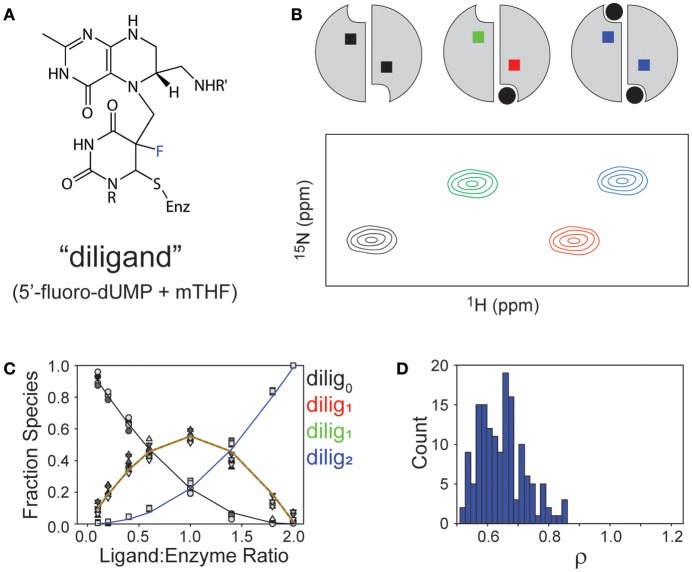
Observations of binding “diligand” to ecTS. **(A)** Chemical structure of stable “diligand” as a model for intermediate II in the reaction mechanism (see Figure 1). The fluorine (blue) is a poor leaving group, which stabilizes this intermediate. **(B)** Schematic of the three liganded states of the TS homodimer (apo, lig_1_, lig_2_) and a resultant “peak quartet.” Peaks are color coded to correspond with the liganded state (black = apo, blue = saturated) and to discriminate bound (red) and empty (green) subunits of the lig_1_ state. **(C)** Fitting of relative K_d_ values from quartet peak intensities, as reported in Sapienza et al. (ref 29). **(D)** Range of ρ values from monte carlo simulations of fit in **(C)**.

As a system for studying sequential ligand binding, TS has a valuable and unique chemical tool to offer. That tool is the combination of 5-fluoro-dUMP (FdUMP) with the normal cofactor mTHF. In the multistep mechanism of TS, after the methylene of mTHF is semi-transferred via a carbon bridge to C5 of dUMP (intermediate II in Figure 1), the H5 proton is the leaving group to yield free oxidized cofactor and the product dTMP tethered via a thiol to C146 of TS. If 5-fluoro-dUMP is used instead, the fluorine is an ineffective leaving group and the reaction becomes trapped at this intermediate (Figure 4A; Stroud and Finer-Moore, 2003). In fact, this is how cancer drug 5-fluorouracil (5-FU) works, by being converted to 5-fluoro-dUMP and becoming an irreversible inhibitor (Hyatt et al., 1997). In this intermediate, the uracil moiety is covalently attached to C146 as well as to cofactor, resulting in a fully covalently linked “diligand” (Stroud and Finer-Moore, 2003; Sapienza et al., 2015). By mixing equal parts FdUMP and mTHF, addition of these reagents to TS results in formation of diligand binding with great preference over separate uncoordinated binding events of FdUMP and mTHF. Most importantly, because of the covalent nature of diligand association, diligand “binding” is in the slow exchange regime on the NMR chemical shift timescale. The importance of slow exchange is that distinct NMR peaks appear for each state that is sampled with sufficient population. Thus, the stepwise progress of diligand binding can be followed quantitatively during an ^1^H/^15^N HSQC-monitored titration of FdUMP and mTHF into TS, and distinct peaks are observed for the singly ligated state (or “lig_1_”; Sapienza et al., 2015). There are a number of TS residue amides for which distinct peaks are observed for apo, singly diligand-bound (dilig_1_) and dilig_2_ TS states that populate during the titration (Figure 4B). Measurement of these various peak intensities can be used to calculate populations during the titrations, and these can be fit using a binding polynomial to determine the relative equilibrium binding association constants for the first and second “diligand binding” processes (Figure 4C). Importantly, this method does not allow fitting of the actual binding constants, but rather, the *relative* binding constants (because there is essentially no free diligand), yielding the cooperativity factor ρ. Carrying out a global fit of these data from four distinct residues yielded a ρ = 0.65 ± 0.08, which indicates a small but measureable degree of negative binding cooperativity for diligand binding to the two protomers of ecTS (Figure 4D; Sapienza et al., 2015). In terms of biological function this small degree of cooperativity is of little consequence, but in terms of quantitative determination of non-cooperative vs. cooperative binding, diligand appears to bind with (a small amount of) negative cooperativity. It should be mentioned that this approach for ρ determination works for small molecule binding that does not affect the intrinsic NMR linewidths. It would be problematic for larger ligands that slow overall tumbling, as simple peak intensity/volume quantitation would be additionally affected by the increase in intrinsic linewidths.

The data above demonstrate that TS exhibits “silent” or “isoergonic” allostery (Fisher and Tally, 1997; Fisher, 2012) with respect to dUMP and cofactor binding. In otherwords, the two binding events have similar affinities, but differ in ΔH, ΔS, and/or ΔC_P_. In the case of diligand, the existence of quartet patterns also reveals allostery in the sense that the structure and/or dynamics of the distal site are affected by binding as read out by the chemical shift. There are few reports of this phenomenon in the literature, likely due to the fact that oligomeric protein ligand binding studies in which the free energies are dissected into entropic and entalpic components, and NMR experiments showing site specific and subunit specific perturbations, are rare. However, we expect silent allostery will be a widespread phenomenon in oligomeric proteins based on a growing body of evidence showing the effects of ligand binding on dynamics as measured by NMR (Clarkson and Lee, 2004; Fuentes et al., 2004; Clarkson et al., 2006; Namanja et al., 2007; Alphonse et al., 2015; Sapienza and Lee, 2016), and the direct linkage between these dynamics and conformational entropy (Frederick et al., 2007; Tzeng and Kalodimos, 2012; Kasinath et al., 2013; Caro et al., 2017). Generally, these dynamic (entropic) effects propogate distally throughout proteins, and will therefore be predicted to propogate across protein interfaces and modulate the conformational entropy of unbound sites. The question then becomes whether this communication is merely incidental or is selected by nature to provide a functional advantage.

## NMR characterization of dimer asymmetry of the lig_1_ state: peak “quartets”

**Figure 5:**
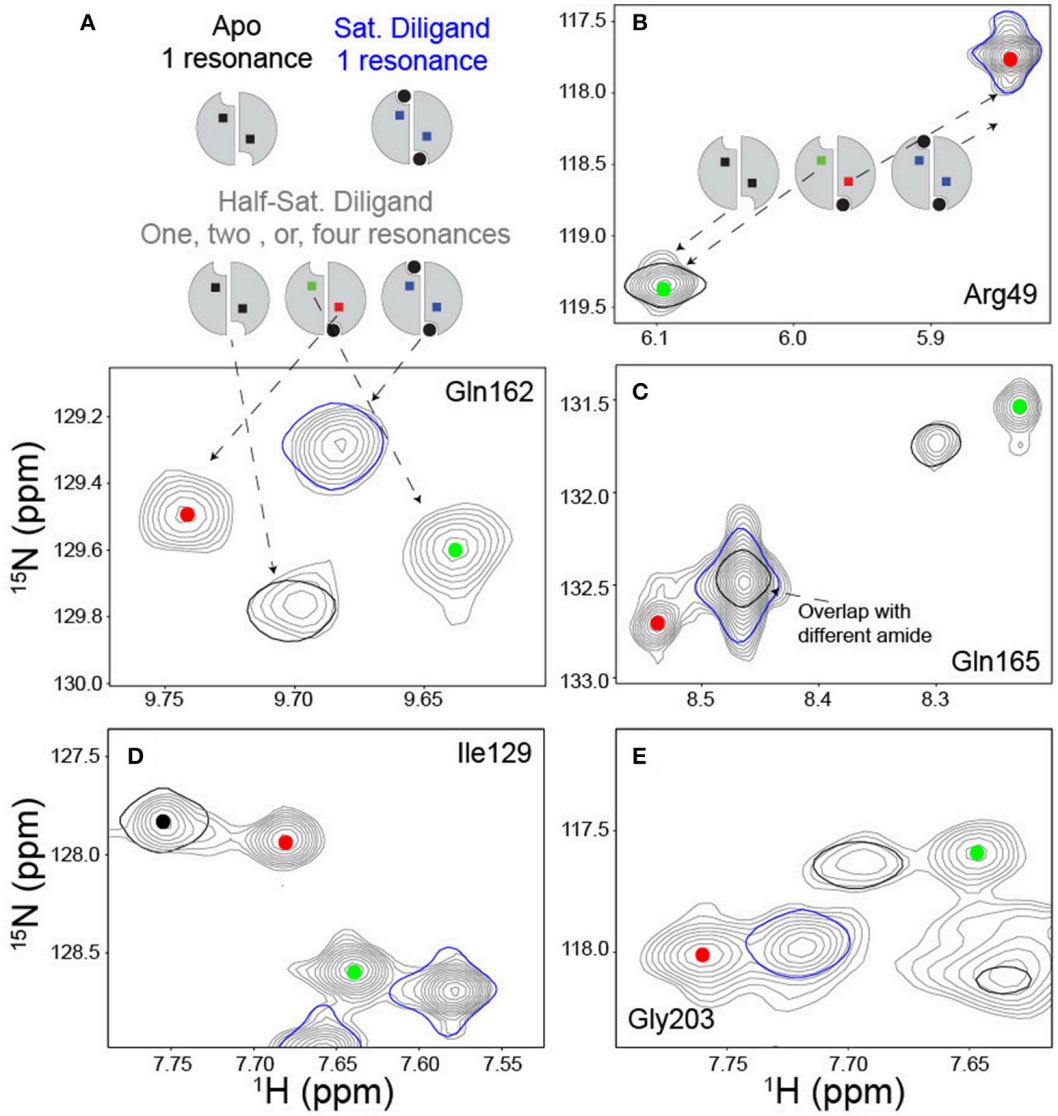
Ligand state peak multiplets in a FdUMP/mTHF (diligand) titration. **(A)** Scheme and spectra of amide with a quartet peak pattern at midpoint of diligand titration. In the scheme, the ligand is shown in black circles and the reporter amide is shown in squares. The titration midpoint spectrum is shown in gray, and the apo and saturated diligand spectra (single contour lines) are shown in black and blue respectively. The red and green dots serve to identify resonances arising from the empty and bound subunits respectively of the singly bound state. **(B)** Example of amide reporter with a doublet peak pattern in which the signal from the empty subunit of the singly bound state overlays with the apo spectrum and the signal from the bound subunit of the singly bound state overlays with the saturated dilagand spectrum. Spectra are colored as described in **(A)**. **(C–E)** Panels provide additional examples of quartet amides and are colored as described above.

The initial NMR work on TS revealed a new and potentially powerful readout of allosteric mechanism in allosteric homo-oligomers (Falk et al., 2016; VanSchouwen and Melacini, 2016), which was first recognized by Freiburger et al. (2011). This was first evident in the diligand (FdUMP + mTHF cofactor) titration described above, that yielded distinct peaks—in slow exchange—for apo, lig_1_, and lig_2_ states of TS (Sapienza et al., 2015). Since the wild-type dimer used in these experiments had both protomers labeled with ^15^N, the lig_1_ species gives rise to two peaks for a subset of residues: one from the residue in the bound protomer, and one from the same residue in the empty protomer (Figure 5A). Of course, there are many residues that show no peak shifting upon diligand binding, and so these remain as single peaks. There are also some residues that show two peak positions (“doublets”), corresponding to free and (fully) bound states, with the singly bound species' peaks overlaying with the expected protomer state (Figure 5B). However, we observed ~50 residues in TS which gave rise to four peaks, or “quartets” (e.g., Figures 5A,C–E), which we also referred to as “ligand state peak multiplets” (Falk et al., 2016). Generally, clear observation of the lig_1_ intermediates in homodimers is elusive, but once observed, these chemical shifts provide the unusual opportunity to measure, experimentally, the effect of ligand occupancy in one protomer on the structural and dynamic features of the other, unbound protomer. Such an influence may reflect key elements of allosteric function. The obvious significance of peak quartets (for homodimers) is that because the residue in the empty protomer has a unique chemical shift, it senses the ligand binding event in the other protomer and has a chemical environment unique from apo, lig_2_, or the same residue in the bound protomer of the lig_1_ state. This forms a basis for communication. For diligand binding to ecTS, we observed ~50 distinct quartets that distribute over a region spanning the diligand binding site and the large intersubunit β-sheet interface. Central residues on this interface lie up to 35 Å from the nearest singly-bound diligand (dilig_1_) atoms. The observation of chemical shift perturbations (CSPs) at these distal sites show the network of residues involved in communication, even though the β-sheet residues are structurally invariant in the various ligation states of ecTS.

Analysis of NMR quartets/ligand state peak multiplets would be much more generally useful if the constraint of slow exchange could be removed. For ecTS, dUMP binding occurs in the fast-intermediate exchange regime, and so the distinct peaks of quartets are not observed from a titration; rather, for the most part, peaks appear to shift as in single ligand binding, and therefore the chemical shifts of lig_1_ states are obscured. Furthermore, unless binding occurs with extreme negative cooperativity, there will be a mixture of liganded states leading to a highly overlapped spectrum (in the limit of slow exchange; in fast exchange the peaks will be averaged according to population weights).

**Figure 6:**
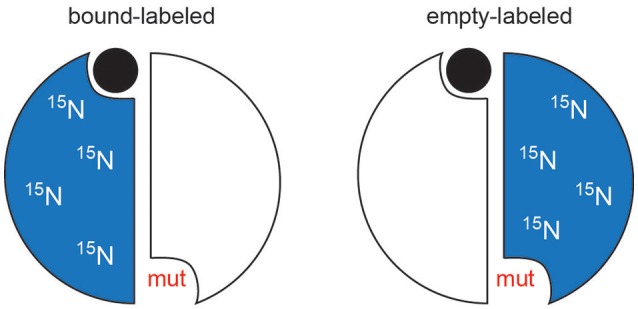
Schematic of mixed labeled dimer (MLD) samples. For simplicity, the mutation is shown in the same protomer as whose active site is compromised. In TS, the mutations are actually made in the opposite protomer's residues (R126 and R127).

To observe the lig_1_ chemical shifts, we constructed a heterodimer composed of one wild-type TS protomer and one mutant (R^126^R^127^ → EE) protomer (Falk et al., 2016). This heterodimer has only one functional active site, which is actually the active site in the mutant since R126 and R127 reside on a loop that interact with dUMP bound to the opposite protomer's substrate binding site (Figure 2). The double charge inversion mutation not only eliminates one binding site, it also allows for chromatographic separation of the heterodimer from the parent wild-type and RREE homodimers. A separation must be performed since the heterodimer is formed from mixing the purified parent homodimers, and a 3-fold mixture results. We separated the heterodimer using anion exchange chromatography (Falk et al., 2016), which has now been further refined to enable separation of up to 10 mg heterodimer in a single run. ITC on the heterodimer showed a *K*_d_ indistinguishable from wild-type TS intrinsic *K*_d,1_ and *K*_d,2_ values. Upon saturation with dUMP, depending on which protomer is labeled with NMR isotopes, the NMR signals from only the bound (RREE mutant ^15^N labeled) or the empty (WT ^15^N labeled) will be observed (Figure 6). Even though four separate samples need to be prepared to observe all four components of peak quartets, this turns out to be an advantage because the lig_1_ forms, referred to as “mixed labeled dimers” (MLDs), can be observed in the absence of the other forms. Comparison of MLDs to the apo spectrum, for example, allows the effect of binding one ligand (dUMP, diligand, or other substrate analog) to the dimer to be observed separately for the two protomers. It also enables the bound and empty protomer peaks to be correctly assigned, which may be ambiguous in many cases. The MLD approach described here breaks degeneracies both in binding, by eliminating apo and lig_2_ species, and in the spectrum, by selective protomer labeling.

The MLD approach was applied to dUMP binding in TS, and in principle can be applied to any stable homodimer. CSPs to the binding and empty subunits were monitored by ^15^N-labeling of the RREE and wild-type protomers, respectively (see above). Monitoring CSPs for the first dUMP binding (dUMP_1_) is done by direct comparison of apo and dUMP_1_-bound MLD. Monitoring CSPs for the second dUMP binding, as well as viewing full quartet patterns, requires reconstruction of the wild-type dUMP_1_ chemical shifts to account for the RREE mutational effects. This was accomplished by simple vector corrections (Falk et al., 2016), and doing so allows for dUMP_1_ chemical shifts to be compared directly to dUMP_2_ chemical shifts, i.e., CSPs for the second dUMP binding event.

**Figure 7:**
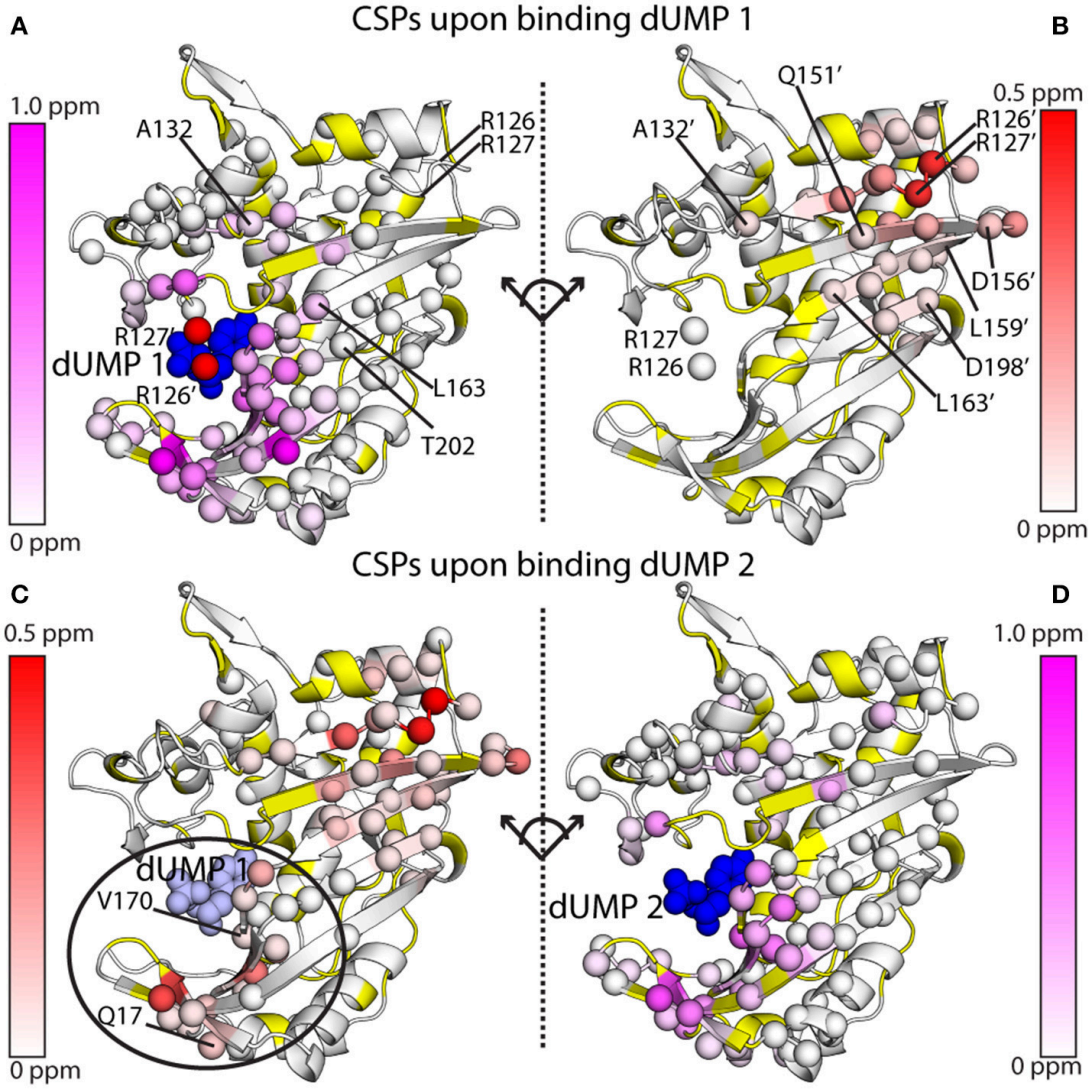
^1^H-^15^N amide chemical shift perturbations (CSPs) for the two dUMP binding events for ecTS, obtained from MLD samples as shown in Figure 6. **(A,B)** CSPs are shown for binding of the first dUMP (blue spheres). CSPs are shown for the binding subunit **(A)** and the empty subunit **(B)**, which are split apart and rotated to take on the same orientation for ease of comparison. Residues from the empty subunit are indicated by primes, and in panel A R126' and R127' from the empty subunit are shown as red spheres, showing how these two residues contact dUMP in the opposite binding site. CSP values are colored according to the scaling scheme shown in each panel. **(C,D)** CSPs are shown for binding of the second dUMP (blue spheres), with the first dUMP shown in light blue spheres. Data are taken from Falk et al. (2016), which also describes how the chemical shifts were corrected and reconstructed for the wild-type peaks to allow calculation of CSPs for the second dUMP.

Before summarizing the effects of dUMP binding, it should be mentioned that the X-ray crystal structures of ecTS show that dUMP binding has a minimal effect on the structure (Perry et al., 1990; Stout et al., 1998) (larger effects are observed upon diligand binding). Thus, the effects observed cannot simply be described as resulting from a distinct conformational change *per se*, but rather result from some combination of very minor, sub-angstrom structural perturbations that spread through the network of interactions (Anderson et al., 1999), as well as long-range transmission of force and dynamical changes to the system that result from dUMP binding. Binding of the first dUMP results in a largely local response of chemical shifts that are restricted to the binding site and minor CSPs out to a few shells. Specifically, CSPs from dUMP_1_ do not extend to the empty dUMP site (Figures 7A,B). By contrast, binding of the second dUMP (based on reconstructed wild-type chemical shifts) has the surprising result of exhibiting CSPs not only locally, but also sizable CSPs that extend to (and beyond) the pre-bound dUMP site (Figures 7C,D). Thus, any dUMP binding cooperativity effects are likely to derive from long-range, cross-interface effects occurring upon binding the second dUMP ligand. These inter-protomer chemical shift effects suggest that there are some structural or mechanical allosteric mechanisms at work in ecTS, even if the thermodynamics of dUMP and diligand binding show allosteric binding at a very subtle level (Falk et al., 2016).

MLD NMR samples of homodimers present a number of opportunities to characterize interprotomer allosteric mechanisms. At the simplest level, qualitative observations of chemical shifts for apo, lig_1_, and lig_2_ states can be made. The unique advantage of MLD samples are that—if binding site suppression is employed in one protomer—they allow direct observation of lig_1_ states by NMR that are very difficult to observe cleanly unless there is extreme negative cooperativity or binding occurs in the slow exchange regime. Because the spectral degeneracy is broken by labeling of only one protomer, distinct properties of the bound and unbound protomers can be monitored. Our initial work on ecTS made such observations of chemical shifts (Falk et al., 2016). Following the elegant work of Mittermaier and coworkers (Freiburger et al., 2011), we noted that peak multiplets from MLD residues enables assessment of allosteric models. For example, MWC-type behavior would show both bound and empty lig_1_ peaks at the position of the lig_2_ peak since the protomers would change conformations in a concerted manner, whereas KNF-type behavior would show the bound lig_1_ peak overlaid with the lig_2_ peak and the unbound lig_1_ peak with apo. To have a large number of residues that report on this microscopic behavior is a powerful tool to assess allosteric mechanism. In ecTS, we observed a mixture of behaviors that were residue dependent (see Figure 6 in Falk et al., 2016). With the selective protomer labeling of MLDs, additional NMR measurements can be made on the bound or empty protomer of the lig_1_ state, such as relaxation measurements for characterization of dynamics, amide hydrogen exchange measurements, or structural measurements such as NOEs, scalar couplings, or residual dipolar couplings (RDCs). One notable application of MLDs would be to gain higher-sensitivity inter-protomer NOEs for structure determination of dimeric species since these have typically been done on labeling mixtures (O'Donoghue et al., 1996; Yang et al., 2010).

**Figure 8:**
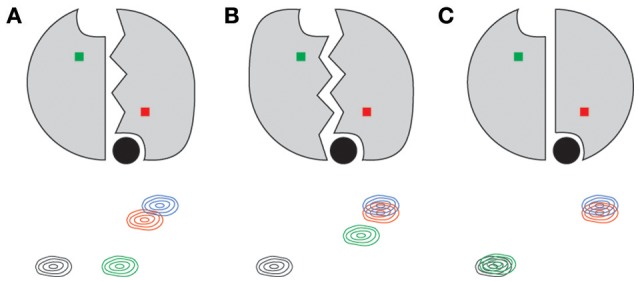
Simple structure-based expectations of quartet peak positions for a symmetric dimer such as thymidylate synthase. Ligand is shown as a filled black circle for each lig_1_ state shown. The residue amide giving rise to the 2D peaks are shown as red and green squares, corresponding to the bound and empty subunits, respectively. Conformational change in a protomer is indicated by jagged lines at the interface. Panels indicate conformational change in the binding protomer only **(A)**, both protomers **(B)**, and in neither **(C)**.

One of the striking qualities of the various chemical shift quartets we observed in ecTS, both for dUMP and diligand binding, is that the quartets exhibit a substantial degree of symmetry in their patterns (Sapienza et al., 2015; Falk et al., 2016; Figure 5). By “symmetry,” we mean that the lig_1_ peak positions are approximately symmetric (pseudosymmetric) with respect to the axis that connects apo and lig_2_ peaks (Figure 4B). Why are these quartets so symmetrical? Because chemical shifts are sensitive reporters of structure, and homodimers can undergo various degrees of structural changes upon ligand binding, we approach this question using several example cases. It should be noted that it is our belief that in order to correctly interpret experimental quartet patterns, it is instructive to first consider the expectations for what quartets would look like using only the simplest of structural considerations for ligand binding of a homodimer. We consider a residue not far from the ligand and near the dimer interface, and we assume in each case that binding of two ligands to the dimer yields a lig_2_ peak that is significantly shifted compared to the apo peak (Figure 8, black and blue peaks). In the first case (Figure 8A), binding of the first ligand induces an overall conformational change in the binding protomer but not in the other. This results in the peak from the bound protomer to be shifted nearly all the way toward the lig_2_ peak, as that site experiences an environment similar to that of lig_2_. The corresponding “distal” peak in the empty protomer would tend to be more apo-like since there is no protomer conformational change, yet it is quite possible that the conformational change in the binding protomer impacts the empty protomer in terms of strain or other propagated forces which results in a modest shift (green peak). Because this altered local environment is considerably different than in the proximal site, the chemical shift of the distal peak would be expected to be unrelated. Hence, in this case there is an expectation for quartet asymmetry. In the second case (Figure 8B), binding of the first ligand induces overall conformational change in both protomers. Thus, this conformational change is expected to have a dominant impact on both proximal and distal sites, and thus the corresponding peaks (red and green) would be expected to be shifted nearly all the way to the lig_2_ peak, with the distal peak (green) perhaps somewhat less shifted since it still has an empty ligand binding site. This quartet pattern is also asymmetric with respect to the apo-lig_2_ axis since the displacement of the green peak from the black peak is much greater than that between red and blue. Finally we consider the third case (Figure 8C) in which there are no conformational changes upon ligand binding. Here the shift upon the first ligand binding is due primarily to the effect of ligand proximity, and with no structural change there is little effect at the distal site. There may be very small chemical shift changes from the apo or lig_2_ peaks for the lig_1_ state in this case, or perhaps the distal peak overlays with apo and the proximal peak overlays with lig_2_, yielding a doublet. The point of these considerations is that none of these scenarios using such simple structural considerations lead to the kind of symmetric quartets that we observe frequently in ecTS (Figure 5 and Falk et al., 2016), and therefore the observed high degree of quartet symmetry is somewhat paradoxical (by these considerations). One possibility is that the lig_1_ chemical shifts reflect dynamic states or modes. If the two protomers are engaged in a global correlated mode of motion, ligand binding may induce compensatory dynamical responses in the two protomers. Another possibility is that the chemical shifts may report on hydrogen-bond strengths, and symmetric quartets result from a give-and-take relationship among symmetrically related sites connected by networks of strain. Deeper investigation will be necessary to have a solid understanding the observed quartet symmetry in ecTS.

## Stabilizing MLDs to protomer reapportionment

NMR analysis of ligand state peak multiplets using mixed labeled dimers has many advantages (VanSchouwen and Melacini, 2016), as discussed above. However, one big caveat is that MLDs will only be effective if the dimer is stable and does not remix to form parent homodimers, otherwise known as reapportionment. Such protomer mixing will dilute the heterodimer and add unwanted signals from the labeled homodimer. Because it is expected that most protein homodimers will exchange on a timescale faster than a few days, it will be important to find ways to “lock” the heterodimer together covalently. One possibility is to use disulfide bonds, although these short linkers may be restrictive to conformational motion and complications can arise if other surface cysteines are present or functionally important. For future applications of MLDs, it will therefore likely become important to develop covalent linker strategies, either utilizing chemistries of natural amino acids or introducing unnatural amino acids capable of biorthogonal chemistry that will allow for selective introduction of covalent linkages.

## Summary

Through initial work on *E. coli* thymidylate synthase (ecTS), we have developed a biophysical and NMR approach to studying intersubunit allosteric substrate binding in protein homodimers. Careful analysis of ITC data that employed global fitting of multiple isotherms was required to reliably determine the binding cooperativity parameter, ρ, for substrate dUMP. Despite half-the-sites reactivity in ecTS, this analysis yielded a ρ of ~1 under standard conditions, although altering conditions or use of other active site ligands showed modest deviation from that value and clearly showed inequivalence of binding thermodynamic parameters (a “touch” of allostery). This inequivalence was corroborated from analysis of NMR ^1^H-^15^N HSQC chemical shifts of the apo (lig_0_), singly liganded (lig_1_, both from the bound and empty promoters), and doubly liganded (lig_2_) states, which showed long-range (35 Å), interprotomer perturbation upon binding the second dUMP. In general, the observation of these homodimer NMR “peak quartets” offers a means to study asymmetric structural and dynamic features of the difficult-to-study lig_1_ state, potentially leading into mechanistic insights into allosteric transmission between protomers. Although in the special case of “diligand” addition (50% titration) to ecTS, HSQC peak quartets could be observed in a single spectrum, most generally quartets will need to be reconstructed using mixed-labeled dimers (MLDs), in which only one protomer can bind ligand and only one protomer is isotope labeled, which will greatly simplify the spectra and allow protomer-specific assignments to peaks. Future work will hopefully address stabilization of MLDs and the ease of their preparation. As for quartets, the pseudo-symmetric disposition of their constituent peaks are fascinating, and the origins of this pseudo-symmetry bears future investigation.

## Author contributions

AL and PS designed the article and both contributed figures. AL wrote the article.

### Conflict of interest statement

The authors declare that the research was conducted in the absence of any commercial or financial relationships that could be construed as a potential conflict of interest.

## References

[B1] Alphonse S. Bhattacharya S. Wang H. Ghose R. (2015). Methyl relaxation measurements reveal patterns of fast dynamics in a viral RNA-directed RNA polymerase. Biochemistry 54, 5828–5838. 10.1021/acs.biochem.5b00828 26333183 PMC5259806

[B2] Anderson A. C. O'Neil R. H. DeLano W. L. Stroud R. M. (1999). The structural mechanism for half-the-sites reactivity in an enzyme, thymidylate synthase, involves a relay of changes between subunits. Biochemistry 38, 13829–13836. 10.1021/bi991610i 10529228

[B3] Benkovic S. J. Hammes-Schiffer S. (2003). A perspective on enzyme catalysis. Science 301, 1196–1202. 10.1126/science.1085515 12947189

[B4] Boehr D. D. McElheny D. Dyson H. J. Wright P. E. (2006). The dynamic energy landscape of dihydrofolate reductase catalysis. Science 313, 1638–1642. 10.1126/science.1130258 16973882

[B5] Caro J. A. Harpole K. W. Kasinath V. Lim J. Granja J. Valentine K. G.. (2017). Entropy in molecular recognition by proteins. Proc. Natl. Acad. Sci. U.S.A. 114, 6563–6568. 10.1073/pnas.1621154114 28584100 PMC5488930

[B6] Clarkson M. W. Gilmore S. A. Edgell M. H. Lee A. L. (2006). Dynamic coupling and allosteric behavior in a nonallosteric protein. Biochemistry 45, 7693–7699. 10.1021/bi060652l 16784220 PMC2453595

[B7] Clarkson M. W. Lee A. L. (2004). Long-range dynamic effects of point mutations propagate through side chains in the serine protease inhibitor eglin c. Biochemistry 43, 12448–12458. 10.1021/bi0494424 15449934

[B8] Eftink M. R. Anusiem A. C. Biltonen R. L. (1983). Enthalpy-entropy compensation and heat capacity changes for protein-ligand interactions: general thermodynamic models and data for the binding of nucleotides to ribonuclease A. Biochemistry 22, 3884–3896. 10.1021/bi00285a025 6615806

[B9] Eisenmesser E. Z. Millet O. Labeikovsky W. Korzhnev D. M. Wolf-Watz M. Bosco D. A.. (2005). Intrinsic dynamics of an enzyme underlies catalysis. Nature 438, 117–121. 10.1038/nature04105 16267559

[B10] Falk B. T. Sapienza P. J. Lee A. L. (2016). Chemical shift imprint of intersubunit communication in a symmetric homodimer. Proc. Natl. Acad. Sci. U.S.A. 113, 9533–9538. 10.1073/pnas.1604748113 27466406 PMC5003262

[B11] Fisher H. F. (2012). Detecting “silent” allosteric coupling. Methods Mol. Biol. 796, 71–96. 10.1007/978-1-61779-334-9_5 22052486

[B12] Fisher H. F. Tally J. (1997). Isoergonic cooperativity in glutamate dehydrogenase complexes: a new form of allostery. Biochemistry 36, 10807–10810. 10.1021/bi9708388 9312269

[B13] Frederick K. K. Marlow M. S. Valentine K. G. Wand A. J. (2007). Conformational entropy in molecular recognition by proteins. Nature 448, 325–329. 10.1038/nature05959 17637663 PMC4156320

[B14] Freiburger L. A. Auclair K. Mittermaier A. K. (2012). Van't Hoff global analyses of variable temperature isothermal titration calorimetry data. Thermochim. Acta 527, 148–157. 10.1016/j.tca.2011.10.018 28018008 PMC5179259

[B15] Freiburger L. A. Baettig O. M. Sprules T. Berghuis A. M. Auclair K. Mittermaier A. K. (2011). Competing allosteric mechanisms modulate substrate binding in a dimeric enzyme. Nat. Struct. Mol. Biol. 18, 288–294. 10.1038/nsmb.1978 21278754 PMC3262843

[B16] Freiburger L. Auclair K. Mittermaier A. (2015). Global ITC fitting methods in studies of protein allostery. Methods 76, 149–161. 10.1016/j.ymeth.2014.12.018 25573261 PMC5182068

[B17] Fritz T. A. Liu L. Finer-Moore J. S. Stroud R. M. (2002). Tryptophan 80 and leucine 143 are critical for the hydride transfer step of thymidylate synthase by controlling active site access. Biochemistry 41, 7021–7029. 10.1021/bi012108c 12033935

[B18] Fuentes E. J. Der C. J. Lee A. L. (2004). Ligand-dependent dynamics and intramolecular signaling in a PDZ domain. J. Mol. Biol. 335, 1105–1115. 10.1016/j.jmb.2003.11.010 14698303

[B19] Henzler-Wildman K. A. Lei M. Thai V. Kerns S. J. Karplus M. Kern D. (2007). A hierarchy of timescales in protein dynamics is linked to enzyme catalysis. Nature 450, 913–916. 10.1038/nature06407 18026087

[B20] Hyatt D. C. Maley F. Montfort W. R. (1997). Use of strain in a stereospecific catalytic mechanism: crystal structures of Escherichia coli thymidylate synthase bound to FdUMP and methylenetetrahydrofolate. Biochemistry 36, 4585–4594. 10.1021/bi962936j 9109668

[B21] Johnson E. F. Hinz W. Atreya C. E. Maley F. Anderson K. S. (2002). Mechanistic characterization of Toxoplasma gondii thymidylate synthase (TS-DHFR)-dihydrofolate reductase. Evidence for a TS intermediate and TS half-sites reactivity. J. Biol. Chem. 277, 43126–43136. 10.1074/jbc.M206523200 12192007

[B22] Kamb A. Finer-Moore J. S. Stroud R. M. (1992). Cofactor triggers the conformational change in thymidylate synthase: implications for an ordered binding mechanism. Biochemistry 31, 12876–12884. 10.1021/bi00166a024 1281428

[B23] Kamerlin S. C. Warshel A. (2010). At the dawn of the 21st century: is dynamics the missing link for understanding enzyme catalysis? Proteins 78, 1339–1375. 10.1002/prot.22654 20099310 PMC2841229

[B24] Kanaan N. Marti S. Moliner V. Kohen A. (2007). A quantum mechanics/molecular mechanics study of the catalytic mechanism of the thymidylate synthase. Biochemistry 46, 3704–3713. 10.1021/bi061953y 17328531

[B25] Kasinath V. Sharp K. A. Wand A. J. (2013). Microscopic insights into the NMR relaxation-based protein conformational entropy meter. J. Am. Chem. Soc. 135, 15092–15100. 10.1021/ja405200u 24007504 PMC3821934

[B26] Lee A. L. (2013). “Chapter is Dynamics and Allostery,” In Enclopedia of Biophysics, ed Roberts G. C. K. (Heidelerg: Springer).

[B27] Lisi G. P. Loria J. P. (2016). Solution NMR spectroscopy for the study of enzyme allostery. Chem. Rev. 116, 6323–6369. 10.1021/acs.chemrev.5b00541 26734986 PMC4937494

[B28] Liu C. C. Richard A. J. Datta K. LiCata V. J. (2008). Prevalence of temperature-dependent heat capacity changes in protein-DNA interactions. Biophys. J. 94, 3258–3265. 10.1529/biophysj.107.117697 18199676 PMC2275698

[B29] Maley F. Pedersen-Lane J. Changchien L. (1995). Complete restoration of activity to inactive mutants of Escherichia coli thymidylate synthase: evidence that *E. coli* thymidylate synthase is a half-the-sites activity enzyme. Biochemistry 34, 1469–1474. 10.1021/bi00005a001 7849005

[B30] Mandel A. M. Akke M. Palmer A. G. (1995). 3rd, Backbone dynamics of Escherichia coli ribonuclease HI: correlations with structure and function in an active enzyme. J. Mol. Biol. 246, 144–163. 10.1006/jmbi.1994.0073 7531772

[B31] Montfort W. R. Perry K. M. Fauman E. B. Finer-Moore J. S. Maley G. F. Hardy L.. (1990). Structure, multiple site binding, and segmental accomodation in thymidylate synthase on binding dUMP and an anti-folate. Biochemistry 29, 6964–6977. 2223754 10.1021/bi00482a004

[B32] Motlagh H. N. Wrabl J. O. Li J. Hilser V. J. (2014). The ensemble nature of allostery. Nature 508, 331–339. 10.1038/nature13001 24740064 PMC4224315

[B33] Nagel Z. D. Klinman J. P. (2009). A 21st century revisionist's view at a turning point in enzymology. Nat. Chem. Biol. 5, 543–550. 10.1038/nchembio.204 19620995

[B34] Namanja A. T. Peng T. Zintsmaster J. S. Elson A. C. Shakour M. G. Peng J. W. (2007). Substrate recognition reduces side-chain flexibility for conserved hydrophobic residues in human Pin1. Structure 15, 313–327. 10.1016/j.str.2007.01.014 17355867

[B35] O'Donoghue S. I. King G. F. Nilges M. (1996). Calculation of symmetric multimer structures from NMR data using a priori knowledge of the monomer structure, co-monomer restraints, and interface mapping: the case of leucine zippers. J. Biomol. NMR. 8, 193–206. 10.1007/BF00211165 22911141

[B36] Perry K. M. Fauman E. B. Finer-Moore J. S. Montfort W. R. Maley G. F. Maley F.. (1990). Plastic adaptation toward mutations in proteins: structural comparison of thymidylate synthases. Proteins 8, 315–333. 10.1002/prot.340080406 2128651

[B37] Phan J. Koli S. Minor W. Dunlap R. B. Berger S. H. Lebioda L. (2001). Human thymidylate synthase is in the closed conformation when complexed with dUMP and raltitrexed, an antifolate drug. Biochemistry 40, 1897–1902. 10.1021/bi002413i 11329255

[B38] Popovych N. Sun S. Ebright R. H. Kalodimos C. G. (2006). Dynamically driven protein allostery. Nat. Struct. Mol. Biol. 13, 831–838. 10.1038/nsmb1132 16906160 PMC2757644

[B39] Rosenzweig R. Kay L. E. (2014). Bringing dynamic molecular machines into focus by methyl-TROSY NMR. Annu. Rev. Biochem. 83, 291–315. 10.1146/annurev-biochem-060713-035829 24905784

[B40] Sage C. R. Michelitsch M. D. Stout T. J. Biermann D. Nissen R. Finer-Moore J.. (1998). D221 in thymidylate synthase controls conformation change, and thereby opening of the imidazolidine. Biochemistry 37, 13893–13901. 10.1021/bi9810510 9753479

[B41] Sapienza P. J. Falk B. T. Lee A. L. (2015). Bacterial thymidylate synthase binds two molecules of substrate and cofactor without cooperativity. J. Am. Chem. Soc. 137, 14260–14263. 10.1021/jacs.5b10128 26517288 PMC4699426

[B42] Sapienza P. J. Lee A. L. (2016). Widespread perturbation of function, structure, and dynamics by a conservative single-atom substitution in thymidylate synthase. Biochemistry 55, 5702–5713. 10.1021/acs.biochem.6b00838 27649373 PMC5604227

[B43] Stevens S. Y. Sanker S. Kent C. Zuiderweg E. R. (2001). Delineation of the allosteric mechanism of a cytidylyltransferase exhibiting negative cooperativity. Nat. Struct. Biol. 8, 947–952. 10.1038/nsb1101-947 11685240

[B44] Stout T. J. Sage C. R. Stroud R. M. (1998). The additivity of substrate fragments in enzyme-ligand binding. Structure 6, 839–848. 10.1016/S0969-2126(98)00086-0 9687366

[B45] Stroud R. M. Finer-Moore J. S. (2003). Conformational dynamics along an enzymatic reaction pathway: thymidylate synthase, “the movie.” Biochemistry 42, 239–247. 10.1021/bi020598i 12525150

[B46] Tzeng S. R. Kalodimos C. G. (2012). Protein activity regulation by conformational entropy. Nature 488, 236–240. 10.1038/nature11271 22801505

[B47] VanSchouwen B. Melacini G. (2016). Cracking the allosteric code of NMR chemical shifts. Proc. Natl. Acad. Sci. U.S.A. 113, 9407–9409. 10.1073/pnas.1611068113 27512035 PMC5003270

[B48] Whittier S. K. Hengge A. C. Loria J. P. (2013). Conformational motions regulate phosphoryl transfer in related protein tyrosine phosphatases. Science 341, 899–903. 10.1126/science.1241735 23970698 PMC4078984

[B49] Wilson P. M. Danenberg P. V. Johnston P. G. Lenz H. J. Ladner R. D. (2014). Standing the test of time: targeting thymidylate biosynthesis in cancer therapy. Nat. Rev. Clin. Oncol. 11, 282–298. 10.1038/nrclinonc.2014.51 24732946

[B50] Yang Y. Ramelot T. A. McCarrick R. M. Ni S. Feldmann E. A. Cort J. R.. (2010). Combining NMR and EPR methods for homodimer protein structure determination. J. Am. Chem. Soc. 132, 11910–11913. 10.1021/ja105080h 20698532 PMC3057626

